# Towards a holistic and solution-oriented monitoring of chemical status of European water bodies: how to support the EU strategy for a non-toxic environment?

**DOI:** 10.1186/s12302-018-0161-1

**Published:** 2018-09-04

**Authors:** Werner Brack, Beate I. Escher, Erik Müller, Mechthild Schmitt-Jansen, Tobias Schulze, Jaroslav Slobodnik, Henner Hollert

**Affiliations:** 1 Department of Effect-Directed Analysis, Helmholtz Centre for Environmental Research UFZ, Permoserstr. 15, 04318 Leipzig, Germany; 2 Department of Ecosystem Analysis, Institute for Environmental Research, ABBt-Aachen Biology and Biotechnology, RWTH Aachen University, Worringerweg 1, 52074 Aachen, Germany; 3 Department of Cell Toxicology, Helmholtz Centre for Environmental Research UFZ, Permoserstr. 15, 04318 Leipzig, Germany; 4 Environmental Toxicology, Center for Applied Geosciences, Eberhard Karls University Tübingen, 72074 Tübingen, Germany; 5 Department of Bioanalytical Ecotoxicology, Helmholtz Centre for Environmental Research UFZ, Permoserstr. 15, 04318 Leipzig, Germany; 6 Environmental Institute, Okruzna 784/42, 97241 Kos, Slovak Republic

**Keywords:** Effect-based methods, Non target analysis, Water Framework Directive, Effect-directed analysis, Integrated assessment

## Abstract

The definition of priority substances (PS) according to the Water Framework Directive (WFD) helped to remove many of these chemicals from the market and to reduce their concentrations in the European water bodies. However, it could not prevent that many of these chemicals have been replaced by others with similar risks. Today, monitoring of the PS-based chemical status according to WFD covers only a tiny fraction of toxic risks, extensively ignores mixture effects and lacks incentives and guidance for abatement. Thus, we suggest complement this purely status-related approach with more holistic and solution-oriented monitoring, which at the same time helps to provide links to the ecological status. Major elements include (1) advanced chemical screening techniques supporting mixture risk assessment and unraveling of source-related patterns in complex mixtures, (2) effect-based monitoring for the detection of groups of chemicals with similar effects and the establishment of toxicity fingerprints, (3) effect-directed analysis of drivers of toxicity and (4) to translate chemical and toxicological fingerprints into chemical footprints for prioritization of management measures. The requirement of more holistic and solution-oriented monitoring of chemical contamination is supported by the significant advancement of appropriate monitoring tools within the last years. Non-target screening technology, effect-based monitoring and basic understanding of mixture assessment are available conceptually and in research but also increasingly find their way into practical monitoring. Substantial progress in the development, evaluation and demonstration of these tools, for example, in the SOLUTIONS project enhanced their acceptability. Further advancement, integration and demonstration, extensive data exchange and closure of remaining knowledge gaps are suggested as high priority research needs for the next future to bridge the gap between insufficient ecological status and cost-efficient abatement measures.

## Background

As stated in the Water Framework Directive “water is not a commercial product like any other but rather, a heritage which must be protected, defended and treated as such” [1]. Chemical pollution is one of the major threats to water quality [2]. However, while European water bodies are contaminated with complex mixtures of ten thousands of chemicals, the chemical status is defined on the basis of 45 priority substances (PS) [3]. Although these compounds have been prioritized according to a thorough and scientifically sound procedure, chemical status as it is defined now covers only a tiny fraction of actual contamination and extensively ignores mixture risks [4]. It may be seen as a success of the establishment of PS that many of these chemicals are no longer permitted for application in the EU and their environmental concentrations are declining. This may be illustrated with the example of pesticides. Out of 20 pesticides on the priority substance list, 13 are banned for application in European agriculture. In a study on Swiss rivers Moschet et al. [5] found among the 50 most frequently detected pesticides only two are priority substances. This means at the same time that 48 out of 50 most frequently detected pesticides are not mandatorily monitored and do not contribute to the establishment of the chemical status. There is evidence that the regulation of individual chemicals as priority substances often results in a replacement with non-regulated substances. This, however, often does not result in reduction of the toxic risk to aquatic biodiversity [6] even if the chemical status appears to have improved. This contradicts the overall ambition of the WFD but also the 7th Environmental Action Programme by the European Commission that proposed a strategic approach for a non-toxic environment [7].

At the same time, even monitoring of the limited set of PS and comparison with Environmental Quality Standards (EQS) leads to a poor chemicals status at 100% of the sites, for example, in Germany [8, 9]. This is caused particularly by ubiquitous chemicals such as mercury, polycyclic aromatic compounds, polybrominated diphenylethers and tributyltin. Unfortunately, abatement options for these legacy chemicals are limited and the categorization of all sites as poor in status according to the one-out–all-out principle indicates a problem (at least one of the EQS values is exceeded) but does not support solutions such as the prioritization of water bodies for action, sources or measures.

Thus, while chemical status assessment along lists of PS is based on a long and successful tradition of regulations and conventions that helped for example phase out many persistent organic pollutants (POPs) [10], it seems to reach its limits today. Reinforcing the chemical status as an indicator for hazardous contamination and as a basis for prioritization and management requires complementation of PS-based status assessment with solution-oriented tools (1) covering chemical contamination and toxic risk in a more integrative and differentiating manner, (2) indicating and diagnosing toxic stress on the ecological status and (3) supporting abatement options to improve the quality status of a water body [11, 12]. These tools should address a much broader range of substances and consider mixture effects and risks rather than concentrations of individual compounds only [4]. Unknown chemicals should be considered as they contribute to risks.

Significant progress in routinely applicable chemical [13] and bioanalytical screening tools [14–16], in effect-directed analysis [17], in platforms supporting the exchange and exploitation of resulting data [18] but also in integrated assessment [19] provides the scientific and technical basis for an extension of chemical status assessment evaluating the degree of convergence to a non-toxic environment as support for prioritization and management. Mixture risk assessment, chemical and toxicological fingerprints based on screening analysis and multi-endpoint effect-based monitoring together with risk- and effect-based trigger (EBTs) values [20, 21] for environmental mixtures have the potential to become routinely applicable tools for monitoring, assessment and diagnosis in near future, and to bridge the gap between the ecological status, toxic contamination and sources thereof and thus management. A chemical status assessment considering mixtures beyond PS is in line with recent suggestions to integrate direct detection of mixture effects, the identification of drivers of toxicity and the definition of priority mixtures as a key to diagnosis and improvement of the quality status of European water bodies [11]. Novel tools for diagnosis of the ecological status including stressor-specific indicators such as pollution-induced community tolerance (PICT) [22], multivariate diagnostic tools [23] assessing stressor-specific responses of microbial communities to pollutants, the SPEcies At Risk index (SPEAR) [24–26] indicating impairment of macroinvertebrate communities due to toxic stress, -omics approaches, next-generation sequencing of organisms exposed or collected in the field [27–29], and eDNA metabarcoding [30–35] may enhance the throughput of structure-based assessment of ecosystems and provide more direct links between chemicals and their modes of action (MoA) and ecosystem functions [36].

**Fig. 1:**
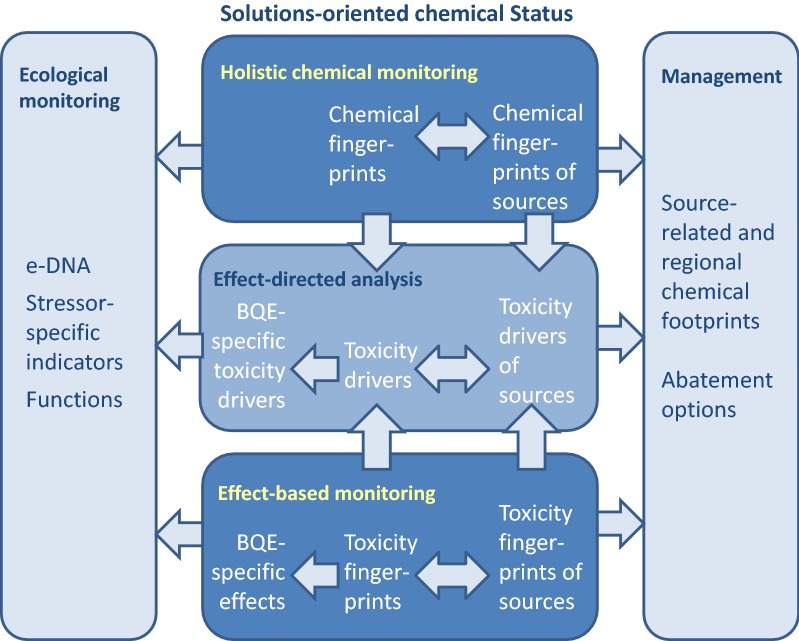
A solution-oriented chemical status that can bridge the gap between the ecological status and management in water bodies impacted by toxic stress, *BQE* Biological Quality Elements

The present paper wants to highlight the new opportunities towards a solution-oriented assessment of an advanced chemicals status that offers links to the ecological status and supports management (Fig. 1). Many of the required tools are available conceptually and as individual approaches in scientific studies, however, they require advancement, integration and demonstration to make them available as monitoring and assessment tools in practice. This paper is meant as a plea to take our claim for a non-toxic environment serious and to perform the science that is required to advance WFD in a way that this claim can be met.

We are aware that the concept of the chemicals status addresses also protection goals beyond toxicity in aquatic ecosystems including human health and secondary poisoning. These goals are not in the focus of the present paper and prioritization of chemicals according to these goals is not touched.

## Advanced chemical monitoring and risk assessment

In Europe, more than 100,000 chemicals are registered in the EU and 30,000–70,000 are in daily use [37], most of them ending up in the environment. Thus, as discussed above, the small set of PS, many of them legacy chemicals, does not reflect the current contamination and the toxic risk to ecosystems and human health in European water bodies. The vast majority of pesticides, biocides, pharmaceuticals, personal care products, surfactants and industrial chemicals as well as mixture risks are ignored. Establishment and monitoring of River Basin-Specific Pollutants (not included in the chemical status) and the EU Watch List mechanism to support prioritization of emerging substances [3] are efforts to address this gap. These attempts are appreciated, however, with 10 new chemicals on the Watch List [38], some of them possibly entering finally in the set of PS, they are probably not sufficient to characterize toxic risks and possible impacts in a holistic and solution-oriented way.

State-of-the-art liquid and gas chromatography coupled to high-resolution mass spectrometry (LC– and GC–HRMS) that allow for screening of hundreds of chemicals at the same time [5, 39] are becoming increasingly available even in routine monitoring laboratories. Automated workflows for screening analyses will significantly enhance the throughput of these methods. Thus, it seems to be not unrealistic that in near future chemical monitoring of surface waters, sediments and biota can include 1000 and more target chemicals with limited additional costs and efforts.

Concentrations as such are of limited relevance for the potential impact on aquatic organisms. However, concentrations can be translated into risk-related values such as hazard quotients (HQs) [19] and toxic units (TUs) [40, 41] based on toxicity to representative organisms, or to potentially affected fraction of species (PAF) [42] based on species sensitivity distributions (SSDs) [43] if data on toxicity are available or can be predicted. The application of mixture effect models such as concentration addition (CA) [44, 45] often used as a default mixture toxicity approach [46] allows for the estimation of an overall risk from the exposure to the measured chemicals represented by ∑TU, ∑HQ or multi-substance (ms)PAF. This approach has been successfully applied for risk assessment and prioritization of pesticides in Swiss streams [5], for the characterization of seasonal dynamics of toxic risks in wastewater treatment plant effluents [41] and for the identification of pesticide mixtures predominating risks in sediments from different European river basins [47]. All these recent studies demonstrate that non-priority substances play a major role for risk and indicate one possible pathway towards a more holistic but also differentiated and solution-oriented complement to current chemical status assessment. Even when the existing classification in good and not good chemical status is kept, ∑HQ, ∑TU or msPAF provide a powerful tool for prioritization of water bodies and chemicals for management.

One of the challenges and research needs is to fill the many gaps in toxicity data of emerging pollutants. In addition to experimental biotesting, in silico tools such as QSAR models and read-across tools [48, 49] may be used to predict toxicity of data-poor chemicals. Pragmatic approaches, considering specific modes of action resulting in excess compared to baseline toxicity [19] may help evaluating and prioritizing sites and water bodies for management. Although there will be no legally binding EQS on the basis of ∑HQ, ∑TU or msPAF for large sets of chemicals and based on uncertain and predicted toxicity data, these values are very helpful to set priorities for further monitoring and abatement. Research should suggest benchmarks of ecotoxicological relevance and linkages to ecological effects. These suggestions need to consider that under environmental conditions, which are typically characterized by multiple stress, effects on aquatic communities can occur at toxicant concentrations that are two or three orders of magnitude below laboratory effect concentrations [26, 50, 51].

Multi-substance assessment as discussed here may be a strong complement to existing approaches and will better inform management decisions. However, it is still based on pre-selected target chemicals. Thus, assessment may strongly benefit from the application of non-target screening (NTS) based on state-of-the-art LC–HRMS and GC–HRMS that is able to record comprehensive chemical fingerprints of complex environmental mixtures and is already applied in routine monitoring such as at the River Rhine, where since 2012 almost 2000 samples have been screened with NTS [52]. Open digital repositories can be used to archive current-state full scan HRMS analysis for retrospective and joint exploitation [53, 54]. At the same time, a bunch of bio-informatics tools including innovative clustering algorithms from metabolomics and genomics including independent component analysis (ICA) and cytoscape/ClueGO pathway analysis [55, 56], hierarchical clustering, *K*-means, self-organizing maps and fuzzy clustering [57, 58] are available and help unravel big and complex analytical datasets. It may be assumed that complex environmental mixtures in water bodies are not just randomly composed but the result of confluence of source-related patterns modified by environmental fate. Considering contamination not only as a mixture of ten thousands of individual chemicals but also as an overlay of chemical fingerprints related to different sources would be a paradigm shift that might strongly impact on chemical monitoring towards a more comprehensive but at the same time simplified approach. It will be of particular value for management by indicating predominating pollution sources impacting on water quality.

As described above, the general technical conditions for more comprehensive chemical monitoring and assessment are extensively fulfilled although the diagnostic power will strongly benefit from future scientific and technological development. To tap the full potential of comprehensive monitoring and chemical fingerprinting significant research is needed. Source-related fingerprints for most relevant anthropogenic sources including different agricultural production, municipal WWTP effluents with different treatment technology, urban and road runoff, industrial production, etc. as well as for natural vegetation need to be compiled and made available as it has been done for natural gas residual fluids from fracturing [59]. The variability of these fingerprints, among sources of the same type, among different seasons or weather conditions or geographical areas needs to be investigated. Performance of NTS on larger sets of source-related and river water samples will also help identify high-intensity signals, ubiquitous vs. rare peaks, components with characteristic isotope patterns of halogens or sulfur, and homologue series [52]. The application of NTS in fate experiments of source-related mixtures under environmental conditions will help understand the impact of partitioning, microbial and photochemical degradation on chemical fingerprints depending on the distance from the source, turbidity, temperature, nutrient concentrations and other system parameters. Relationships between intensities of source-specific peaks with different transformation rates in a given river system might even help estimate the residence time of a mixture in the water package and thus the distance to the source. NTS may be also applied to internal contamination in biota. The comparison of internal with external fingerprints may provide good indications for bioaccumulating compounds and metabolites.

Although the overlay of source-related fingerprints might cover a significant fraction of chemical signals detected in water bodies integrating over many different sources, understanding the patterns from known sources will also help to identify unique site specific and newly emerging compounds from so-far unknown sources. This will substantially help prioritize peaks for identification efforts. In the River Rhine NTS monitoring, this approach has been demonstrated and resulted in the identification of compounds occurring in erratic peak events such as 2-phenyl-2-(2-piperidinyl)acetamide and tetracarbonitrile-1-propene from specific production sites [52].

## Effect-based monitoring for toxicity fingerprints

While advancement of chemical monitoring and mixture assessment has been discussed above, the requirement to link chemical to ecological status strongly calls for complementing chemical with effect-based monitoring using in vitro, in vivo and in situ effect-based methods (EBMs) [14]. In the European subgroup chemical monitoring and emerging pollutants (CMEP) within the Common Implementation Strategy (CIS) for the WFD, a specific task was established for the development of a technical report on effect-based tools. According to the mandate from the CMEP, the aim of the report was to identify potential effect-based tools (e.g. bioassays, biomarkers and ecological indicators) that could be used in the context of the different monitoring programmes (surveillance, operational and investigative) linking chemical and ecological status assessments and to be used as suggestion for the revision of the WFD [60]. EBMs detect groups of chemicals exhibiting a common effect on biota, and detected effects are believed to provide particularly useful data for hypothesis generation and correlation with ecological observations. Toxicity fingerprints may be understood as the differentiated outcome of monitoring with a battery of in vitro and in vivo EBMs conceptually linked to short- and long-term effects on aquatic organisms including representatives for the WFD Biological Quality Elements (BQE) algae, invertebrates and fish. In situ approaches and community-based indicators and diagnostic tools should be used to bridge these findings to effects on the BQEs. EBMs cover all chemicals contributing to these effects including knowns and unknowns, regulated and non-regulated substances. Thus, if major toxicological endpoints and modes of action (MoA) are covered, EBMs may help describe the chemical status in a more holistic way and provide a link to the ecological status. Further, EBMs are believed to support the diagnosis of causes for impairment of ecosystems and thus help find abatement options [11, 60].

For implementation in practice, EBMs should meet major practical requirements of monitoring, diagnosis and assessment. This includes low volumes of test systems and low amounts of samples required, high throughput, robustness against matrix effects, the selective and sensitive detection of toxicants affecting important endpoints and the relevance for adverse effects on aquatic organisms, populations and communities [16, 60]. A comprehensive set of in vitro assays seems to be an ideal tool with respect to throughput, volume requirements and diagnostic power. Within the NORMAN Network on Emerging Pollutants and the Solutions EU project a draft of a common position on how to use EBMs for water quality monitoring is recently under development (i.e. methodology to define effect-based trigger values; recommendations for a common battery of bioassays; quality/performance criteria for the benchmarking of bioassays) [14–16, 19–21, 54, 61–63] and will be suggested directly to the EBM working group within the Common Implementation Strategy (CIS) for the WFD. In a recently published European-wide proof-of-principle study, the reliability of EBMs for screening of endocrine disrupting compounds was analyzed to harmonise monitoring and data interpretation methods, and to contribute to the current WFD review process. Water and wastewater samples were collected across Europe and analysed using chemical analyses and EBMs. The study demonstrated that the inclusion of effect-based screening methods into monitoring programmes for estrogens in surface waterbodies would be a valuable complement to chemical analysis [15, 21].

Although great progress on the use of EBMs has been achieved within the last 5 years, there are also challenges and shortcomings. For example, Busch et al. [19] identified approximately 100 modes of action aggregated into 31 categories when screening almost 1000 chemicals frequently detected in the aquatic environment for available MoA information. Only for a minor portion of these MoAs specific EBMs are readily available. In particular, a lack of EBMs covering neurotoxicity and behavior was identified (cf. Busch et al. [19]). On the other hand the EBM typically applied in test batteries for water quality assessment cover the major responses, that a multiplexed assay covering of 25 nuclear receptors (NR) and 48 transcription factors (TF) identified as relevant for diverse types of water [64]. At the same time our understanding is still limited how the MoAs translate into effects at the organism, population and community level and how mixture components with different MoAs interact. The further development of adverse outcome pathways (AOP) [65] and particularly mixture AOPs is required to reduce these knowledge gaps [66]. Since currently MoA-based EBMs are not able to cover the full range of effects, apical tests on organisms representing the BQEs are required as a basis of a test battery complemented with in vitro MoA-based EBMs covering long-term effects such as endocrine disruption, genotoxicity and neurotoxicity, which are not addressed by short-term apical tests [14]. Effect-based monitoring will help reduce the bias towards well-known historical burden chemicals in monitoring and consider all chemicals affecting the selected biological systems. To this end, a solution-oriented chemical status including effect-based monitoring and confirmation on the community level using approaches like SPEAR or PICT [67] will help indicate if the improvement of the ecological status needs to consider pollution management. Effect-based monitoring is also believed to be a powerful tool for control of success of management measures. For example, the efficacy of advanced treatment methods such as ozonation and activated charcoal, as well as the risk of transformation products can be identified using EBMs [62, 68–74]. For categorizing water quality effect-based trigger (EBT) values are required. In a first attempt, such values have been developed on the basis of existing Environmental Quality Standards (EQS) [20, 21, 61, 75].

A set of more than 100 EBMs has been successfully tested for benchmarking organic micropollutants in 10 waste, recycled and drinking waters [64]. Batteries of EBMs have been applied in the framework of the project SOLUTIONS [11, 12] in the Danube River basin [76, 77] as well as in small streams in Switzerland [62]. In vitro EBMs for estrogenic receptor-mediated effects have been thoroughly validated for screening of endocrine disruptors in surface and waste waters [78] and artificial mixtures [63]. In situ tools like the PICT-approach have been successfully applied to demonstrate effects of complex mixtures on ambient communities [79] and the success of restoration efforts in WWTPs [80]. These studies clearly showed the potential of EBMs and are a very good basis for larger demonstration efforts involving environmental and water agencies and monitoring practitioners. These efforts need to involve the demonstration of state-of-the-art sampling and enrichment technology [81, 82] as well as the integration of chemical screening of priority and emerging chemicals to allow for a direct comparison and to demonstrate the power of integrated monitoring.

Further research needs to include the extension of available effect-based monitoring tools towards a larger set of MoAs that are applicable to complex environmental mixtures. A risk-based prioritization of groups of chemicals sharing common MoAs is required but also an increased understanding of processes along AOPs also for mixtures. It should be considered that different molecular initiating events may culminate in the same AOP [83]. As an example, endocrine disruption may be the result of nuclear receptor binding as a molecular initiating event but also of impacts on steroid synthesis [69]. Both converge into the key event of endocrine disruption and the adverse outcome of impaired reproduction. Thus, despite joint effects being expected from both chemicals on the endocrine system of an organism, these cannot be detected in bioassays based solely on interaction with nuclear receptors.

Combinations of EBMs may also be a powerful diagnostic tool to establish unique toxicity fingerprints of source-related mixtures of chemicals [4] and may help together with chemical fingerprints in the development of hypotheses on sources of contamination. To better understand the impact of pollution sources on water quality in rivers and lakes and on potential adverse effects environmental monitoring should start to compile characteristic toxicity and chemical fingerprints of major sources of water pollution such as effluents from municipal WWTPs with different treatment technologies, effluents for major industrial branches, land use-specific agricultural runoff, urban runoff and natural vegetation types. An early example provides the heat map in a benchmarking study across diverse water types [64]. This will help link patterns derived by effect-based monitoring of surface waters to probable sources and thus to management options.

## Effect-directed analysis of drivers of toxicity

Neither EBMs nor chemical analysis alone provide direct links between exposure to chemicals and effects. Overall toxicity as quantified by whole-organism tests and apical endpoints as well as adaptive stress responses may be triggered by complex mixtures of contaminants involving many substances. In contrast, specific effects involving unique receptors in the organism such as endocrine disruption, photosynthesis inhibition or inhibition of specific enzymes are often caused by few individual drivers of mixture toxicity. The identification of these drivers is key to the decision on targeted and cost-efficient abatement options and requires the integration of effect-based monitoring with chemical analytical tools [17].

In cases where candidate chemicals for a specific endpoint together with quantitative data on effective potencies are available, mass balance approaches based on the model of concentration addition (CA) [84] applying Biological Equivalent Concentrations (BEQs) [77], iceberg modelling [20] or Toxic Units [40] are an efficient way to assess the contribution of these candidates to the overall activity. This approach has been quite successfully applied to endocrine disruptors binding to nuclear receptors in cell-based assays [77], to alterations on steroidogenesis [85], and to photosynthesis inhibition [62]. Increasing availability of effective data, for example, from high-throughput toxicity screening in ToxCast [86, 87] and the enhanced accessibility particularly via the CompTox Chemistry Dashboard [88] will further improve the application of mass balance approaches. However, in vivo and in vitro toxicity data gaps together with the large amounts of unknown chemicals still hamper the application of mass balances, which are based on having a comprehensive set of candidate toxicants. Analyzing Swiss WWTP effluents Schymanski et al. [89] found that only 1.2% of the detected peaks were assigned to the 376 target compounds that have been addressed. The rest were unknowns.

Effect-directed analysis (EDA) and related approaches combining biotesting with chemical analysis and chromatographic fractionation for reducing complexity of the mixtures has been applied for decades in drug discovery and environmental research [90–95] and further advanced recently [17]. This approach has demonstrated its analytical power in numerous studies on water and sediments using different toxicological endpoints including different types of endocrine disruption [85, 96–103], mutagenicity [104–106], dioxin-like effects [107–109] and effects on daphnids and algae [110] as well as in wastewater treatment evaluations [102, 103]. For the identification of site-specific drivers of toxicity, this approach is the most powerful tool so far and the only approach that directly provides cause–effect relationships. Community-based tools such as PICT have been demonstrated to be an ideal complement to EDA confirming cause–effect relationships in situ, on a higher level of biological organization [79, 111]. At the moment, EDA is still relatively costly and laborious and thus not a tool for routine monitoring but helps identify emerging hazardous chemicals and thus new substances for monitoring at hot spots of pollution identified, for example, by effect-based monitoring. The development of high-throughput EDA techniques has started [112, 113] and will further enhance the application of these tools in future.

However, also large-scale techniques are required linking chemical and effect-based monitoring in a way that, at least, good hypotheses on toxicity drivers (e.g. on a catchment scale) can be developed. Multivariate analysis of chemical and toxicity data on larger sets of environmental samples provides such a possibility to link non-target analytical data including the many peaks representing unknowns with effect-based monitoring data. This approach was generated more than a decade ago introducing the term virtual EDA [114, 115]. Instead of chromatographic separation of the samples into toxic and non-toxic fractions, a virtual fractionation using multivariate statistics such as partial least square analysis (PLS) separates chemical signals into those co-varying with measured effects and those without co-variance. Recently, this approach has been taken up and successfully applied for the identification of diaminophenazines from dye production in river water impacted by effluents from an industrial wastewater treatment plant [116, 117]. However, a rigorous evaluation of virtual EDA and the criteria for its applicability are still missing although this approach could be a milestone in linking effects to chemical signals at larger scales, where the application of classical EDA is too costly and time consuming.

## Links to managements: from diagnostic fingerprints to evidence-based chemical footprints

Chemical footprints have been introduced as a promising method to express ecotoxic impacts from chemical emissions as the dilution needed to avoid freshwater ecosystem damage [118, 119]. Chemical footprints may be defined for individual products [120] or for whole cities or countries [118]. Emission-based chemical footprints are derived from chemical emission inventories and use species sensitivity distributions (SSDs) to define “safe” concentrations. The derivation of chemical footprints is described as a procedure with several steps [121] involving among others the estimation of emissions and of their ecological impact as msPAF and the quantification of natural boundaries derived from food web models [122] or boundaries defined by policies. This approach has been applied for prospective mixture risk assessment for river catchments with diverse land uses [123]. The study could show the significant impact of land use and demonstrate that the exposure scenarios could be associated with predicted species losses under certain circumstances [123].

Emission-based chemical footprints involving much more chemicals than the WFD priority pollutants and considering mixture effects may provide enormous progress compared to typical WFD assessment. However, the approach very much relies on the existence, availability and validity of emission data or estimates thereof and is still limited to known chemicals with known toxicities, while source-related fingerprints and environmental mixtures are often composed from tens of thousands of chemicals. Thus, emission-based footprint derivation should be complemented with footprints that are derived from real-world complex mixtures represented by chemical and toxicological fingerprints as described above. Validation of emission-based footprints with footprints based on source-related fingerprints will help anchor predictions in real world contamination and provide information on the relevance of unknown or ignored chemicals emitted; for example with municipal and industrial wastewater effluents or urban and agricultural runoff. This will support more targeted and efficient management and reduce the chemical footprint on our way to a non-toxic environment.

## Conclusions and research needs

Approaches and methods for a more holistic and solution-oriented monitoring including chemical screening informing risk assessment, effect-based methods, effect-directed identification of toxicity drivers as well as chemical footprints as tools for assessment, prioritization and management are conceptually available and have been tested, for example, in the SOLUTIONS project. To deliver EC’s requirements of achieving and maintaining a good chemical and ecological status in European rivers and lakes, targeted and cost-efficient management is required demonstrating, advancing and evaluating these tools together with monitoring practitioners. In case studies on different scales, the new methods should be integrated with ecological monitoring developing monitoring strategies that allow for a direct interlink between chemical and ecological status and that make use of promising advanced methods for diagnosing stress on aquatic communities such as SPEAR, PICT and e-DNA. Open access European data exchange platforms and advanced multivariate data evaluation tools should be further developed to deal with the enormous sets of chemical analytical, bioanalytical and ecological data the novel screening tools deliver and should be applied to support monitoring on different scales and demonstrated in the case studies. Substantial research is required to unravel chemical and toxicological fingerprints for major anthropogenic sources, as well as backgrounds patterns from natural soils and vegetation in water resources and to translate this information into indicators relevant for prioritization and management such as chemical footprints.
